# Radiographic changes and clinical outcomes after open and closed wedge high tibial osteotomy: a systematic review and meta-analysis

**DOI:** 10.1186/s13018-019-1222-x

**Published:** 2019-06-14

**Authors:** Xiangyun Cheng, Fanxiao Liu, Fei Xiong, Yijiang Huang, Alexander Christoph Paulus

**Affiliations:** Department of Orthopaedic Surgery, Physical Medicine and Rehabilitation, University Hospital of Munich (LMU), Campus Großhadern, Marchioninistrasse 23, 81377 Munich, Germany

**Keywords:** CWHTO, OWHTO, Radiographic results, Clinical outcomes, Osteoarthritis

## Abstract

**Background:**

The purpose of this meta-analysis is to examine changes in radiological variables and clinical outcomes between open and closed wedge high tibial osteotomy (OWHTO and CWHTO, respectively), which have ongoing controversial issues in numerous quantitative clinical studies.

**Methods:**

PubMed, Embase, and the Cochrane Library were systematically searched for suitable controlled trials between Jan 1, 1999, and Feb 2, 2018. The inclusion criteria included studies written in English, studies with a level of evidence of I–IV, and studies presenting comparisons between OWHTO and CWHTO. The main clinical and radiographic results were extracted and pooled using Stata 12.0.

**Results:**

After searching for and screening trials, 28 trials involving 2840 knees were eligible for the meta-analysis. After OWHTO or CWHTO, clinical scores, including the American Knee Society Score, Hospital for Special Surgery Knee Score, Lysholm score, and Visual Analog Scale pain score, improved (*p* < 0.05), but the range of motion was unchanged (*p* > 0.05). The anatomical femorotibial angle (SMD 0.04, 95% CI − 0.66 to 0.74) and hip-knee-ankle angle (SMD 0.11, 95% CI − 0.11 to 0.33) data suggested that the OWHTO and CWHTO groups were similar in function of correction. Posterior tibial slope increased (SMD − 0.71, 95% CI − 1.04 to − 0.37) after OWHTO but decreased (SMD 0.72, 95% CI 0.35 to 1.08) after CWHTO. OWHTO decreased patellar height (*p* < 0.05), while patellar height did not change significantly after CWHTO (*p* > 0.05).

**Conclusion:**

This meta-analysis indicates that compared with CWHTO, OWHTO increases the posterior slope, decreases the patellar height, and provides a similar accuracy of correction; however, CWHTO leads to a decreased posterior slope and an unchanged patellar height. Therefore, programs should be personalized and customized for the specific situation of each patient.

## Background

The load distribution at the physiological tibiofemoral joint is usually inconsistent [1, 2] because the load of the medial compartment accounts for more than 60% of the joint load as a result of varus malalignment [3]. Therefore, the medial compartment is susceptible to lesions in the early stages of osteoarthritis, and this susceptibility is partial caused by the additional force [4].

High tibial osteotomy (HTO) is now considered effective surgical treatments for medial compartment arthritis [5, 6], which can maintain the integrity of the knee, reduce pain, and extend joint life, and HTO has been gaining attention in recent years [7]. The principle of high tibial osteotomy is to correct varus limb alignment so that the excessive pressure is transferred from the medial compartment to the relatively healthy lateral compartment.

Medial open-wedge (OW) and lateral closed wedge (CW) HTO, during which a wedge-shaped cut is made in the medial and lateral parts of the tibia [8], respectively, and internal fixation with a plate and screws is then performed at the end [2, 9], are two of the most commonly used surgical methods among young and highly active patients.

To evaluate these two surgical methods, numerous studies have compared the radiographic changes and clinical results after OWHTO and CWHTO [6, 10–14]. The choice of osteotomy site, anatomical femorotibial angle (AFTA), hip-knee-ankle angle (HKA), leg length, patellar height, posterior tibial slope, and correction accuracy are among the ongoing controversial issues [15–18]. As individual studies may not be able to provide sufficient data on their own, the effect of OWHTO and CWHTO should be assessed objectively using pooled analysis.

Although several meta-analyses [14, 19, 20] were performed earlier, most evaluated only postoperative outcomes. A preoperative comparative analysis is also quite important, which could make results more persuasive and accurate. The posterior tibial slope (PTS) was measured by two different methods (measuring line: posterior tibial cortex or tibial mechanical axis) in some studies comparing OWHTO and CWHTO [16, 18, 21–24], but some meta-analyses [14, 19, 20] pooled the results of different methods together, which may be not completely accurate. Additionally, numerous recently published studies [23–27] have longer follow-up times or present different directions for evaluating the effects of these two surgical methods. Therefore, regarding the current dilemma, the purpose of this meta-analysis is to examine changes in radiological variables and clinical outcomes between OWHTO and CWHTO which have ongoing controversial issues in numerous quantitative clinical studies and to assist surgeons in determining the appropriate method according to the patient condition. This study hypothesizes that OWHTO is better than CWHTO in clinical outcomes, that there are no differences in the function of correction between OWHTO and CWHTO, that posterior tibial slope increases after OWHTO and decreases after CWHTO, and that patellar height decreases after OWHTO and increases after CWHTO.

## Methods

This meta-analysis was performed in strict accordance with the Preferred Reporting Items for Systematic Reviews and Meta-Analyses (PRISMA) Statement [28].

### Search strategy

PubMed, Embase, and the Cochrane library were searched systematically, using English, to identify relevant studies published between Jan 1, 1999, and Feb 2, 2018. The complete search terms used in these three databases were tibial osteotomy [All Fields] AND high tibial osteotomy [All Fields] OR open wedge osteotomy [All Fields] OR open tibial osteotomy [All Fields] OR closed wedge high tibial osteotomy [All Fields] OR closed wedge osteotomy [All Fields] OR closed tibial osteotomy [All Fields]. Additionally, manual searches utilizing the reference lists of in included studies were performed to obtain articles neglected by searching the databases as mentioned above.

### Assessment of study eligibility

The research question and eligibility criteria were determined a priori. The inclusion criteria were (1) studies presenting a comparison of results between OWHTO and CWHTO, (2) studies presenting at least one result for radiographic and clinical outcomes, and (3) clinical studies written in English and with a level of evidence of I–IV. Postoperative indicators less than 1 year of follow-up were divided into subgroups for analysis to reduce heterogeneity. For multiple articles containing the same sample population, the one with the relatively larger sample size or longer follow-up time was included in our investigation, and the others were used as a reference.

The exclusion criteria were (1) studies such as case reports, book chapters, review articles, summaries of experience, and cadaver studies; (2) animal or cell studies; (3) studies involving participations who had rheumatoid arthritis or former infection in the knee; and (4) studies presenting data from original articles that cannot be expressed as the mean ± standard deviation.

### Data extraction

The following information was extracted from each study: the first author’s surname; publication year; country of origin; participant characteristics (number, age, and gender); operated knees; trial duration; type of internal fixation; clinical outcomes, including range of motion (ROM), Hospital for Special Surgery knee score (HSS), American Knee Society Score (KSS), Visual Analog Scale pain score (VAS), Lysholm score and the total of the Western Ontario and McMaster University osteoarthritis (WOMAC) index; and radiographic results, including anatomical femorotibial angle (AFTA), hip-knee-ankle angle (HKA), mechanical axis deviation (MAD), mechanical medial proximal tibia angle (MMPTA), posterior tibial slope angle (PTSA), Caton-Deschamps index (CDI), Insall-Salvati Index (ISI), and Blackburne-Peel index (BPI). All processes of data extraction were performed by two reviewers (Xiangyun Cheng & Fanxiao Liu) repeatedly. Any discrepancies reached a consensus by discussion with an arbitrator (Fei Xiong).

### Quality assessment

Quality assessment was performed for each included study using the “assessing risk of bias” table [29] for randomized controlled trial (RCT) studies and using the MINORS (Methodological Index for Nonrandomized Studies) checklist for nRCT articles. Discrepancies were resolved through discussion among the researchers (Xiangyun Cheng, Fanxiao Liu and Fei Xiong). Additionally, the quality of evidence for radiographic results and clinical outcomes was evaluated using the Grading of Recommendations Assessment, Development, and Evaluation (GRADE) approach [20].

### Statistical analysis

For data measurement, the standardized mean difference (SMD) with a 95% confidence interval (CI) was pooled using Stata 12.0 (Stata Corp, College Station, TX, USA). According to the Cochrane Handbook, statistical homogeneity, clinical homogeneity, and methodological homogeneity were evaluating using *I*^2^ [28, 29]. If *p* > 0.1 and, *I*^2^ < 50%, considering the heterogeneity is small, a fixed effects model was performed. Otherwise, the causes of heterogeneity were investigated using subgroup analysis and sensitivity analysis [14]. If the cause could not be identified, a random effects model was used. Subgroup analyses were performed based on the preoperative and postoperative results of the two groups (OWHTO and CWHTO), the data from RCTs and nRCTs, follow-up (more than 1 year). Kappa values were used to evaluate the degree of agreement between the two authors as follows: fair 0.40 to 0.59, good 0.60 to 0.79, and excellent 0.8 or more [29].

## Results

### Results of the search and study selection

**Fig. 1:**
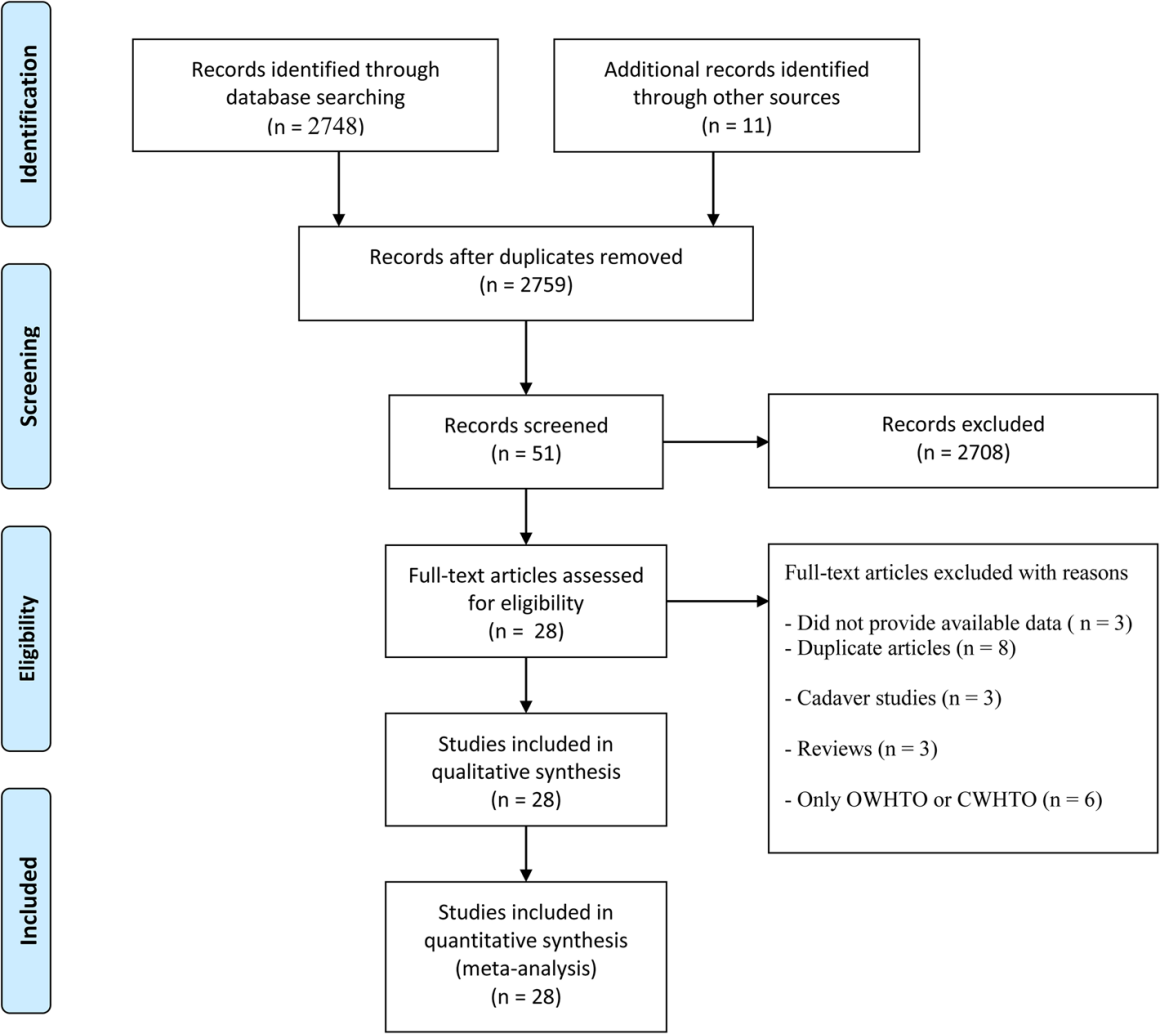
Flow diagram of included and excluded studies

After screening the titles and abstracts, 2708 of 2759 records (2748 retrieved from the 3 databases and 11 identified from references) were excluded. Subsequently, after downloading and identifying the full text, 3 articles with unavailable data, 8 duplicates, 3 cadaver studies, and 3 reviews were excluded (Fig. 1). Eventually, 28 articles [10–13, 15–18, 21–27, 30–42] published between 1999 and 2018 met the inclusion criteria and were included in the meta-analysis.

### Basic characteristics of included studies

The basic information of the included studies is summarized in Table 1. Of these articles, eight articles [10, 11, 13, 24, 26, 27, 30, 31] were randomized controlled trials (RCTs) and 20 articles [12, 15–18, 21–23, 25, 32–42] were nRCTs. Two studies [27, 30] used the WOMAC score, and only one study used the Lysholm score [13]. The duration of follow-up in these studies ranged from 6 months to 13 years. These studies included a total of 2788 participants (1370 in OWHTO and 1438 in CWHTO) involving 2840 knees (1384 in OWHTO and 1456 in CWHTO).

**Table 1:** The main information of the included RCT and prospective studies

Author	Year	Country	Study design	Surgery	Person	M	F	Knee	Age mean ± SD (range)	Fixation type	BMI	Time range	Follow-up (year)
Polat	2017	Turkey	R	C	29	NA	NA	29	45.5 ± 5.1	Puddu plate	NA	1990–2010	13.9 ± 6.2
				O	88	NA	NA	88	44.6 ± 7.4	Staples	NA	1990–2010	11.7 ± 5.4
Kim	2016	Korea	RCT	C	30	10	20	30	54.1 ± 4	Stepped plate	25.7 ± 3.4	03.2013–05.2015	1
				O	30	9	21	30	54.3 ± 3.8	TomoFix plate	24.4 ± 3.2	03.2013–05.2016	1
Nerhus	2015	Norway	RCT	C	35	NA	NA	35	30–60	Two staples	NA	2007–2013	0.5
				O	35	NA	NA	35	30–60	Puddu plate	NA	2007–2014	0.5
Duivenvoorden	2015	Netherlands	R	C	354	203	151	354	49.4 ± 9	TomoFix plate/Puddu plate	29.5 ± 5.8	1993–2012	10.6 ± 5.1
				O	112	72	39	112	48.7 ±10.1	TomoFix plate/Puddu plate	28.5 ± 4.5	1993–2012	7.4 ± 3.2
Portner	2014	Canada	R	C	18	15	3	18	46.5 ± 5.17 (36–54)	Two staples	NA	2006–2012	05
				O	26	20	6	26	43.9 ± 8.48 (21–59)	Plate and screws	NA	2007–2010	0.5
Hanada	2014	Japan	R	C	20	6	14	16	63.2 (30–78)	NA	NA	09.2005–01.2007	1
				O				10	63.2 (30–78)	NA	NA	2007–2009	1
Egmond	2014	Netherlands	RCT	C	25	16	9	25	50.3 ± 7.4	Four-hole angle stable plate	28.4 ± 3.0	2002–2013	7.9
				O	25	15	10	25	47.1 ± 8.5	Phosphate plate	29.7 ± 4.2	2002–2013	7.9
Duivenvoorden-2	2014	Netherlands	RCT	C	45	27	18	45	49.5 ± 9.2	Staples	28.2 ± 4.9	01.2001–04.2004	6
				O	36	24	12	36	49.9 ± 7.9	Puddu plates	27.3 ± 5.4	01.2001–04.2005	6
Deie	2014	Japan	R	C	12	3	9	12	57.8 ± 6.0	Plate fixation	24.8 ± 3.3	2011–2012	1
				O	9	3	6	9	57.5 ± 6.0	Plate fixation	28.2 ± 4.0	2011–2012	1
Tabrizi	2013	Iran	R	C	16	12	4	21	35.1 ± 9.7	LorT-plate	NA	NA	0.5
				O	16	13	3	21	36.5 ± 8.1	One plate	NA	NA	0.5
Soleimanpour	2013	Iran	R	C	16	12	4	21	35.1 ± 9.7	LorT-plate	NA	NA	0.5
				O	16	13	3	21	36.5 ± 8.1	Plate	NA	NA	0.5
Bae	2013	Korea	P	C	74	4	70	78	58.75 ± 7.5	Mini-plate-staple	25 ± 2.5	04.2005–01.2007	3.4
				O	28	2	25	30	56.3 ± 7.5	Puddu plate	25.1 ± 2.7	04.2005–01.2007	3.4
Filho	2013	Germany	P	C	117	60	57	117	54.5 ± 9.7	NA	27.8 ± 5.1	NA	13.2 ± 6.2
				O	24	16	8	24	57.3 ± 7.0	NA	29.4 ± 5.2	NA	7.4 ± 4.7
Amzallag	2013	Germany	P	C	97	205	116	97	49.7 ± 10.30	NA	27 ± 4.4	01.2008–03.2009	0.5
				O	224			224	53.6 ± 8.6	NA	28 ± 5	01.2008–03.2009	0.5
Song	2012	Korea	R	C	50	12	38	50	60.1 (46–65)	Two staples	NA	01.1996–03.2006	3.6
				O	50	10	40	50	57.9 (49–65)	Plates	NA	01.1996–03.2006	3.4
Ducat	2012	France	P	C	97	205	116	92	52 ± 9	NA	27.0 ± 4.4	01.2008–03.2009	0.5
				O	224			210	49.7 ± 10.3	NA	28.6 ± 5.5	01.2008–03.2009	0.5
Magnussen	2011	France	R	C	30	21	9	30	59 (45–72)	Blade and screws	NA	01.2006–12.2009	1
				O	32	22	10	32	54 (42–65)	TomoFix plate	NA	01.2006–12.2009	1
Hankemeier	2010	Germany	P	C	26	42	19	26	53 (27–74)	Screw-plate fixation	NA	01.2001–12.2005	2.2
				O	35			35	44 (18–68)	TomoFix plate	NA	01.2001–12.2005	2.2
Gaasbeek	2010	Netherlands	RCT	C	25	16	9	25	48.4 ± 8	Four-hole locked plate	28.4 ± 2.9	01.2003–03.2005	1
				O	25	14	11	25	49.8 ± 7.4	Four-hole locked plate	29.7 ± 4.2	01.2003–03.2005	1
EI-Azab	2010	Germany	R	C	50	6	538	50	45.8 ± 4	L-plate	NA	01.2000–12.2006	0.7–1.25
				O	50			50	44.6 ± 5	TomoFix plate	NA	01.2000–12.2006	0.7–1.25
Luites	2009	Netherlands	RCT	C	19	27	15	19	53 (40–68)	TomoFix plate	< 30	12.2001–07.2004	2
				O	23			23	53 (40–68)	TomoFix plate	< 30	12.2001–07.2004	2
Schaefer	2008	Switzerland	R	C	29	18	40	29	47 (26–65)	Plate fixation	26.7 (19–32.9)	1996–2002	2
				O	29			29	46 (26–64)	Puddu plate	26.5 (18–32.8)	1996–2002	2
Raaij	2008	Netherlands	P	C	8	NA	NA	8	50 ± 8.6	Two staples	NA	2006	1
				O	28	NA	NA	28	50 ± 8.1	Puddu plate	NA	2006	1
EI-Azab	2008	Germany	R	C	50	79	31	60	45 ± 5.7	L-plates	NA	01.2000–12.2006	0.7
				O	60			60	47.2 ± 3.6	Puddu plate	NA	01.2000–12.2006	0.7
Brower	2006	Netherlands	RCT	C	47	27	20	47	50.8 (22–64)	Two staples	28 (19–47)	01.2001–04.2004	1
				O	45	32	13	45	49.6 (21–67)	Puddu plate	28.2 (21–40)	01.2001–04.2004	1
Hoell	2005	Germany	R	C	51	32	19	51	52.1 ± 8.4	Coventry staples	29 ± 4.2	2001	0.8–3
				O	40	28	12	40	46.4 ± 8	Puddu plate	30 ± 5.2	2001	0.8–3
Brouwer-2	2005	Netherlands	RCT	C	24	12	12	24	47.7 ± 7.4	Two staples	NA	01.2001–01.2003	1
				O	26	20	6	26	50.1 ± 8.2	Puddu plate	NA	01.2001–01.2003	1
Tigani	2001	Italy	R	C	44	22	22	47	59.1 ± 15	Coventry staples	NA	NA	3.6
				O	34	12	24	40	63.1 ± 7	Plaster cast	NA	NA	3.6

*NA* not available, *RCT* randomized controlled trial, *R* retrospective, *P* prospective, *C* closed, *O* open, *M* male, *F* female

### Risk of bias

**Fig. 2:**
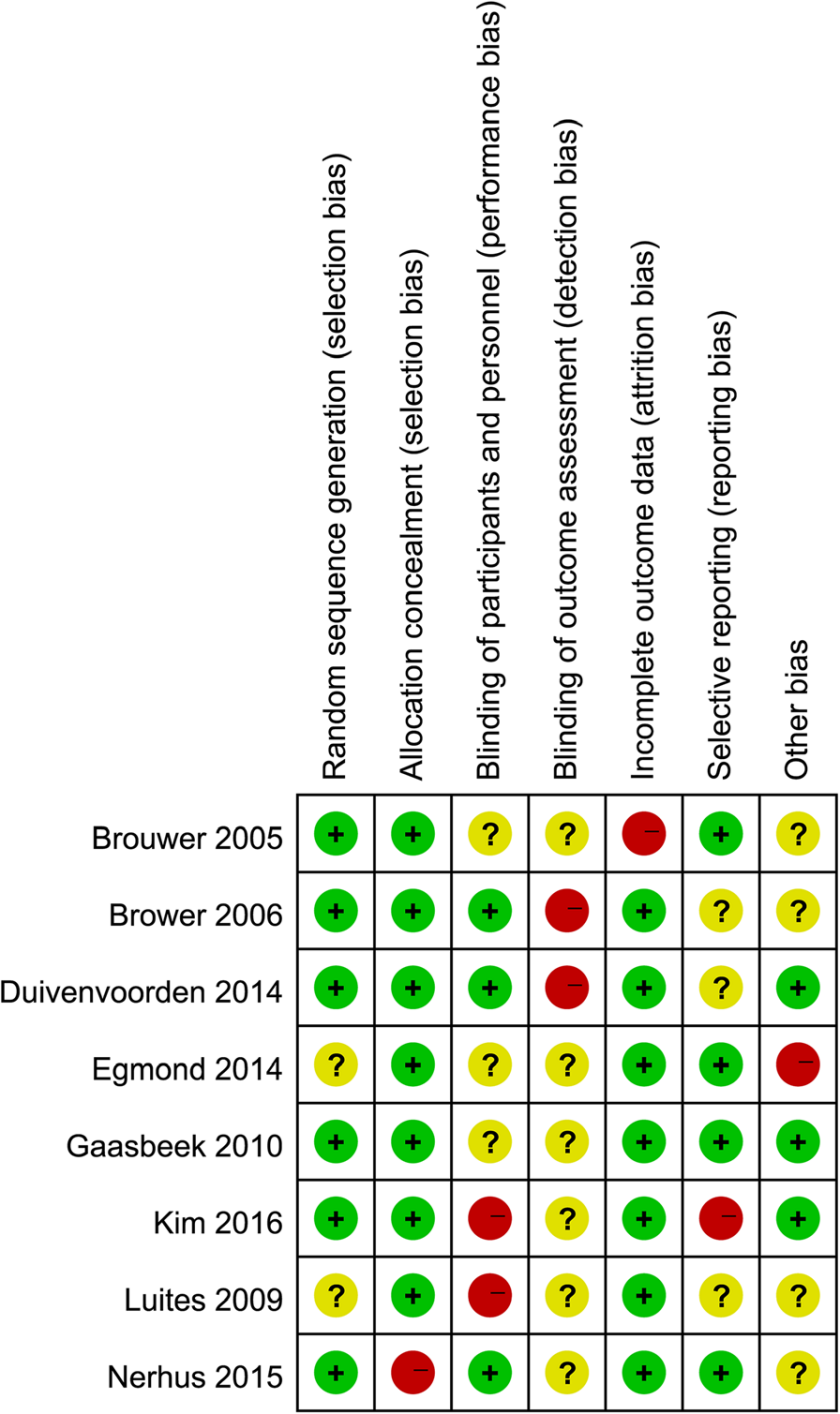
Risk of bias of included in randomized controlled trials. +, no bias; –, bias; ?, bias unknown

The risk of bias, which is used to assess RCTs, is presented in Fig. 2. The MINORS score assessing nRCTs was 15 ± 2, and their levels of evidence were III or IV (Table 2). The kappa values regarding the evaluation of the risk of bias in RCTs and nRCTs were 0.821 and 0.803, respectively, indicating the excellent degree of agreement between the two researchers (Xiangyun Cheng & Fanxiao Liu).

**Fig. 3:**
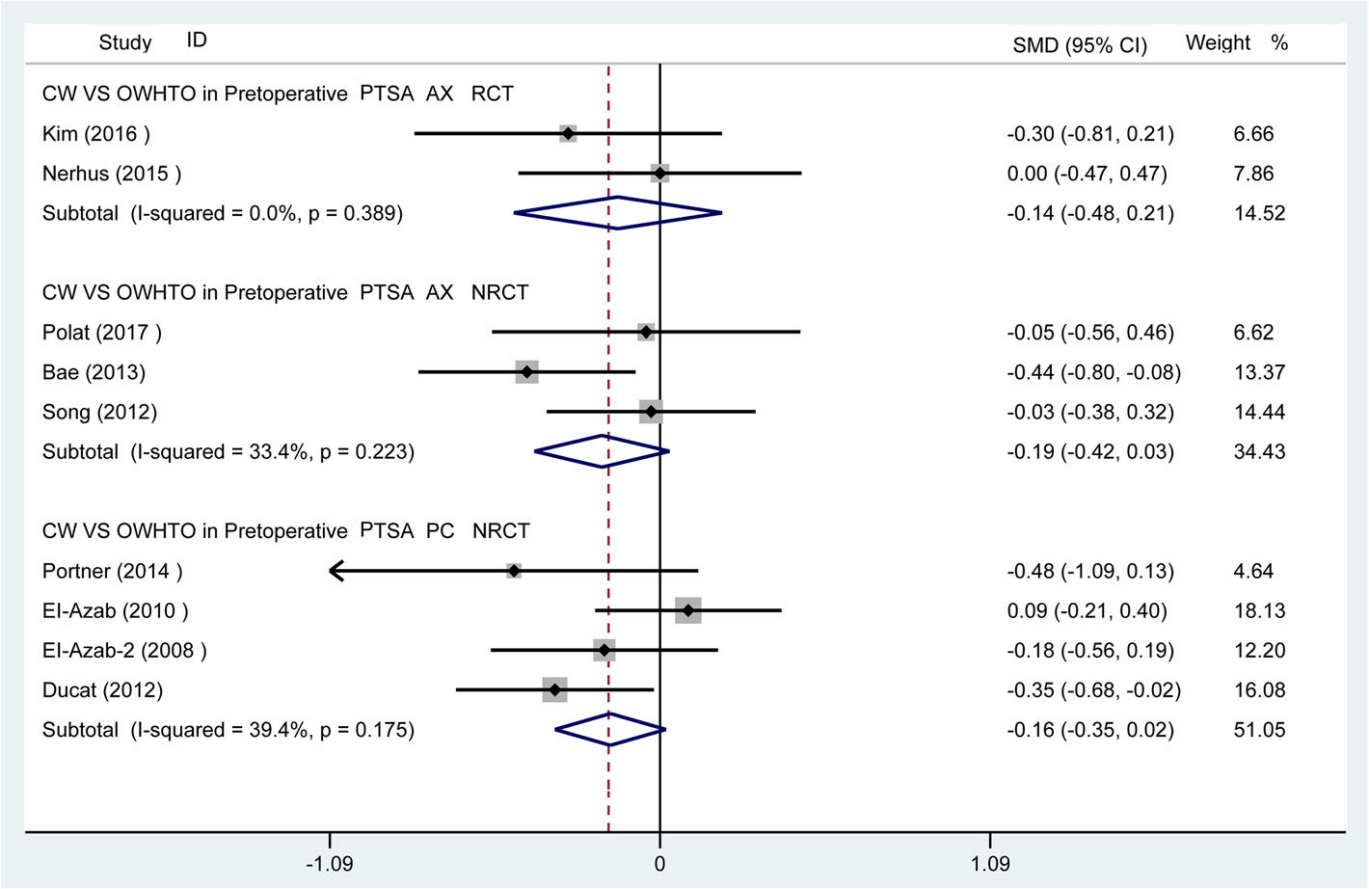
Preoperative comparison between two groups for PTS

**Fig. 4:**
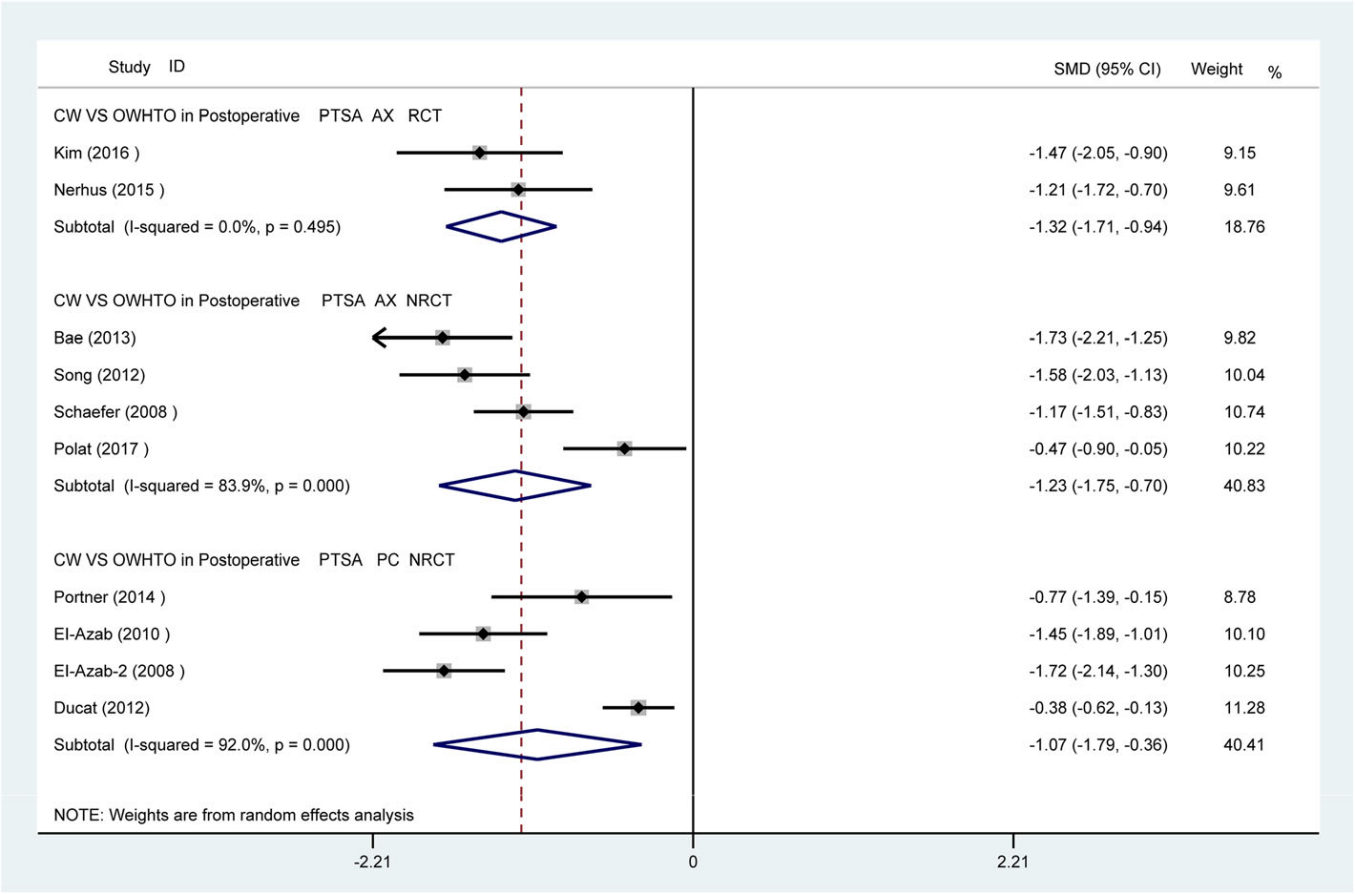
Postoperative comparison between two groups for PTS

**Table 2:** **The MINORS score of the relevant studies (NRCT)**

Author	Year	Country	Study design	No. of groups	LoE	MINORS score	Follow-up (year)
Polat	2017	Turkey	Retrospective	2 (OW/CW)	3	13	12.4 ± 6.2
Duivenvoorden	2015	Netherlands	Retrospective	2 (OW/CW)	3	13	10.6 ± 5.1
Portner	2014	Canada	Retrospective	3(OW/CW/CO)	3	14	0.5
Hanada	2014	Japan	Retrospective	2 (OW/CW)	4	12	1
Deie	2014	Japan	Retrospective	2 (OW/CW)	3	15	1
Tabrizi	2013	Iran	Retrospective	2 (OW/CW)	4	12	0.5
Soleimanpour	2013	Iran	Retrospective	2 (OW/CW)	4	12	0.5
Bae	2013	Korea	Prospective	2 (OW/CW)	3	17	3.4
Filho	2013	Germany	Prospective	2 (OW/CW)	3	15	13.2 ± 6.2
Amzallag	2013	Germany	Prospective	2 (OW/CW)	3	17	0.5
Song	2012	Korea	Retrospective	2 (OW/CW)	3	18	3.6
Ducat	2012	France	Prospective	2 (OW/CW)	3	14	0.5
Magnussen	2011	France	Retrospective	2 (OW/CW)	3	16	1
Hankemeier	2010	Germany	Prospective	2 (OW/CW)	3	15	2.2
EI-Azab	2010	Germany	Retrospective	2 (OW/CW)	3	19	0.7–1.25
Schaefer	2008	Switzerland	Retrospective	2 (OW/CW)	3	15	2
Raaij	2008	Netherlands	Prospective	2 (OW/CW)	3	15	1
EI-Azab	2008	Germany	Retrospective	2 (OW/CW)	3	16	0.7
Hoell	2005	Germany	Retrospective	2 (OW/CW)	4	15	0.8–3
Tigani	2001	Italy	Retrospective	2 (OW/CW)	3	18	3.6

*OWHTO* open wedge high tibial osteotomy, *CWHTO* closed wedge high tibial osteotomy, *CO* combined osteotomy, *nRCT* non-randomized controlled trial, *LoE* level of evidence, *MINORS* methodological index for non-randomized controlled studies

### Clinical outcomes

Preoperative HSS, VAS, and KSS were not statistically significant between the OWHTO and CWHTO groups (*p* > 0.05). Similarly, postoperative comparisons of the two groups showed no statistically significant differences (*p* > 0.05). However, for these three indicators, the postoperative scores were significantly better than the preoperative scores. The postoperative ROM difference between the two groups was not statistically significant (*p* > 0.05). OWHTO increased the length of the leg, while CWHTO decreased the condition (*p* < 0.05).

### Radiological outcomes

Comparison of the AFTA between the CWHTO and OWHTO groups showed no statistically significant difference (*p* > 0.05) either at preoperation (SMD 0.04, 95% CI − 0.66 to 0.74**)** or postoperation (SMD 0.08, 95% CI − 0.23 to − 0.37**)** (Table 3). However, the postoperation AFTA was better than the preoperation AFTA (*p* < 0.05) for both the CWHTO and OWHTO groups, which demonstrated that the OWHTO and CWHTO surgeries were similar in function of correction. Furthermore, similar results of the H-K-A angle between the two methods were found (Table 4).

**Table 3:** Radiological and clinical results of the preoperative comparison and postoperative comparison between OWHTO and CWHTO

Categories and comparison	Studies	Mean difference		*P* value	Heterogeneity	
		Mean	(95% CI)		*I*^2^ (%)	*p* value
CWHTO VS OWHTO in preoperative outcomes						
Radiological outcomes RCT						
HKA	10, 24,26,27,30	0.11	(− 0.11 to 0.33)	0.33	0	0.792
MAD	26	0	(− 0.47 to 0.47)	0.90	NA	NA
MMPTA	26	0	(− 0.46 to 0.46)	0.95	NA	NA
PTSA (AX)	24,26	− 0.14	(− 0.48 to 0.21)	0.38	0	0.39
CDI	27,30	0.01	(− 0.39 to 0.37)	0.18	21	0.43
ISI	26,31	0.02	(− 0.33 to 0.38)	0.89	49	0.04
BPI	31	0.49	(0.05 to 1.06)	0.03	NA	NA
Radiological outcomes nRCT						
HKA	17,21,33,36,39,41	− 0.08	(− 0.28 to 0.12)	0.44	0	0.85
MAD	23	0.34	(− 0.08 to 0.74)	0.12	NA	NA
MMPTA	23	0.06	(− 0.36 to 0.48)	0.79	NA	NA
PTSA (AX)	16,21,23	− 0.19	(− 0.42 to 0.03)	0.09	34.4	0.23
PTSA (PC)	12,18,22,38	− 0.16	(− 0.35 to 0.02)	0.08	39.4	0.18
CDI	15,38,42	− 0.01	(− 0.21 to 0.20)	0.95	94	0.001
ISI	22,38	− 0.20	(− 0.53 to 0.13)	0.23	0	0.43
BPI	21,36,38	− 0.22	(− 0.50 to 0.06)	0.13	0	0.37
AFTA	22,33,40	0.04	(− 0.66 to 0.74)	0.09	76.6	0.014
Clinical outcomes RCT						
ROM	24	0.08	(− 0.42 to 0.59)	0.75	NA	NA
HSS	2,10,11	− 0.04	(− 0.294 to 0.22)	0.78	0	0.92
KSS	24	0.15	(− 0.35 to 0.66)	0.55	NA	NA
WOMAC	27,30	− 0.32	(− 0.72 to 0.07)	0.12	0	0.99
VAS	10,13,24,27,30,31	− 0.11	(− 0.32 to 0.10)	0.23	4.2	0.39
Clinical outcomes nRCT						
ROM	21	0.28	(− 0.62 to 0.03)	0.08	NA	NA
HSS	21,23	− 0.10	(− 0.39 to 0.19)	0.39	0	0.49
KSS	23,36	− 0.05	(− 0.35 to 0.26)	0.76	0	0.47
Lysholm score	13	− 0.34	(− 0.95 to 0.27)	0.28	NA	NA
CWHTO VS OWHTO in postoperative outcomes						
Radiological outcomes RCT						
HKA	10, 24,26,27,30	0.21	(− 0.01 to 0.44)	0.06	0	0.83
HKA CORRECTION	24,26,27	0.16	(− 0.13 to 0.46)	0.28	0	0.65
MAD	26	0.45	(− 0.03 to 0.92)	0.065	NA	NA
MMPTA	26	− 2.76	(− 3.42 to − 2.10)	0.001	NA	NA
PTSA (AX)	24,26	− 1.32	(− 1.71 to − 0.94)	0.001	0	0.495
CDI	27,30	0.82	(0.18 to 1.46)	0.012	59	0.547
ISI	26,31	0.46	(0.09 to 0.82)	0.014	0	0.55
BPI	31	0.49	(0.05 to 1.06)	0.03	NA	NA
Radiological outcomes nRCT						
HKA	17,21,33,36,39,41	− 0.04	(− 0.24 to 0.15)	0.67	0	0.71
HKA CORRECTION	17	0.12	(− 0.39 to 0.63)	0.64	NA	NA
MAD	23	0.32	(− 0.11 to 0.74)	0.14	NA	NA
MMPTA	23	− 1.98	(− 2.34 to − 1.62)	0.001	NA	NA
PTSA (AX)	16,21,23	− 1.19	(− 1.40 to − 0.99)	0.001	83.9	0.001
PTSA (PC)	12,18,22,38	− 0.85	(− 1.04 to − 0.67)	0.001	92	0.001
CDI	15,38,42	0.79	(0.55 to 1.03)	0.001	30	0.24
ISI	22,38	0.06	(− 0.27 to 038)	0.74	0	0.99
BPI	21,36,38	0.33	(0.04 to 0.70)	0.05	58.1	0.09
AFTA	22,33,40	0.08	(− 0.23 to 0.39)	0.46	12.2	0.32
Clinical outcomes RCT						
VAS	10,13,24,27,30,31	− 0.11	(− 0.31 to 0.10)	0.30	33.8	0.18
ROM	24	− 0.19	(− 0.70 to 0.32)	0.46	NA	NA
HSS	10,24,31	0.12	(− 0.21 to 0.45)	0.49	38.4	0.20
KSS	24	0.41	(− 0.01 to 0.93)	0.11	NA	NA
WOMAC	27,30	− 0.42	(− 0.12 to 0.18)	0.09	13	0.346
Clinical outcomes nRCT						
Leg length change	16,37	− 1.33	(− 1.68 to − 0.97)	0.001	84.6	0.01
ROM	43	− 0.36	(− 0.66 to 0.03)	0.06	NA	NA
HSS	21,23	− 0.09	(− 0.38 to 0.20)	0.55	0	0.45
KSS	23,31	0.09	(− 0.22 to 0.39)	0.58	0	0.52
Lysholm score	13	− 0.38	(− 0.99 to 0.24)	0.23	NA	NA

Postoperative results were compared between the two groups in the lower half of the table

*AX* Anatomical axia, *PC* posterior cortex, *RCT* randomized controlled trial, *nRCT* non-randomized controlled trial, *NA* not available, *ROM* range of motion, *HSS* Hospital for Special Surgery Knee Score. *KSS* American Knee Society Score, *VAS* Visual Analog Scale Pain Score, *WOMAC* the total of Western Ontario and McMaster University Osteoarthritis Index, *AFTA* anatomical femorotibial angle, *HKA* hip-knee-ankle angle, *MAD* mechanical axis deviation, *MMPTA* mechanical medial proximal tibia angle, *PTSA* posterior tibial slope angle, *CDI* Caton-Deschamps Index, *ISI* Insall-Salvati Index, *BPI* Blackburne-Peel index

**Table 4:** Radiological and clinical results comparing preoperative and postoperative outcomes in OWHTO or CWHTO group

Categories and comparison	Studies	Mean difference		*p* value	Heterogeneity	
		Mean	(95% CI)		*I*^2^ (%)	*p* value
Preoperative VS postoperative results in CWHTO						
Radiological outcomes RCT						
HKA	10, 24,26,27,30	− 3.17	(− 3.74 to − 2.60)	0.001	79	0.001
MAD	26	− 2.31	(− 2.92 to − 1.70)	0.001	NA	NA
MMPTA	26	− 2.90	(− 3.58 to − 2.23)	0.001	NA	NA
PTSA (AX)	24,26	0.72	(0.35 to 1.08)	0.001	4.8	0.31
CDI	27,30	− 0.11	(− 0.50 to 0.29)	0.59	0	0.59
ISI	26,31	− 0.10	(− 0.46 to 0.26)	0.58	0	0.84
BPI	31	− 0.18	(− 0.75 to 0.39)	0.53	NA	NA
Radiological outcomes nRCT						
HKA	17,21,33,36,39,41	− 2.75	(− 3.77 to − 1.73)	0.001	95	0.001
MAD	23	− 6.20	(− 7.46 to -4.95)	0.001	NA	NA
MMPTA	23	− 1.54	(− 2.13 to − 0.95)	0.001	NA	NA
PTSA (AX)	16,21,23	0.35	(0.13 to 0.58)	0.015	28.8	0.24
PTSA (PC)	12,18,22,38	0.56	(0.20 to 0.92)	0.003	68.8	0.003
CDI	15,38,42	− 0.13	(− 0.33 to 0.07)	0.19	48.9	0.19
ISI	22,38	− 0.03	(− 0.33 to 0.28)	0.69	0	0.87
BPI	22,36,38	0.06	(− 0.22 to 0.34)	0.69	74.4	0.05
AFTA	22,33,40	− 3.19	(− 3.54 to − 2.69)	0.001	0	0.73
Clinical outcomes RCT						
VAS	10,13,24,27,30,31	1.66	(1.05 to 2.27)	0.001	84.7	0.001
ROM	24	− 0.11	(− 0.61 to 0.40)	0.68	NA	NA
HSS	2,10,11	− 1.06	(− 1.34 to − 0.79)	0.001	84	0.002
KSS	24	− 1.61	(− 2.19 to − 1.02)	0.001	NA	NA
WOMAC	27,30	1.65	(1.19 to 2.11)	0.001	54	0.14
Clinical outcomes nRCT						
Lysholm	13	− 1.40	(− 2.16 to − 0.69)	0.001	NA	NA
ROM	21	− 0.15	(− 0.54 to 0.24)	0.45	NA	NA
HSS	21,23	− 0.09	(− 0.38 to 0.20)	0.55	0	0.45
KSS	23,36	− 1.68	(− 2.72 to − 0.63)	0.002	90.6	0.52
Preoperative VS postoperative results in OWHTO						
Radiological outcomes RCT						
HKA	10, 24,26,27,30	− 2.78	(− 3.12 to − 2.45)	0.001	91.4	0.001
MAD	26	− 1.95	(− 2.52 to − 1.38)	0.001	NA	NA
MMPTA	26	− 2.76	(− 3.42 to − 2.10)	0.001	NA	NA
PTSA (AX)	24,26	− 0.59	(− 0.94 to − 0.23)	0.001	0	0.68
CDI	27,30	0.65	(0.25 to 1.06)	0.002	0	0.45
ISI	26,31	0.28	(0.08 to 0.64)	0.04	0	0.34
BPI	31	0.58	(0.03 to 1.14)	0.04	NA	NA
Radiological outcomes nRCT						
HKA	17,21,33,36,39,41	− 2.84	(− 3.14 to − 2.55)	0.001	93.3	0.001
MAD	23	− 7.03	(− 7.83 to − 6.24)	0.001	NA	NA
MMPTA	23	− 1.98	(− 2.35 to − 1.62)	0.001	NA	NA
PTSA (AX)	16,21,23	− 0.71	(− 1.04 to − 0.37)	0.001	69.5	0.02
PTSA (PC)	12,18,22,38	− 0.24	(− 0.39 to − 0.09)	0.002	42.7	0.17
CDI	15,38,42	0.56	(0.40 to 0.72)	0.001	39.9	0.001
ISI	22,38	0.18	(− 0.14 to 0.50)	0.26	25.8	0.25
BPI	21,36,38	0.92	(0.63 to 1.21)	0.001	29.6	0.23
AFTA	22,33,40	− 3.04	(− 3.41 to − 2.67)	0.001	39	0.19
Clinical outcomes RCT						
VAS	10,13,24,27,30,31	1.65	(1.03 to 2.26)	0.001	84.6	0.001
ROM	24	− 0.35	(− 0.86 to 0.17)	0.18	NA	NA
HSS	10,24,31	− 1.52	(− 2.16 to − 0.88)	0.009	89.6	0.001
KSS	24	− 2.14	(− 2.78 to − 1.50)	0.001	NA	NA
WOMAC	27,30	1.22	(0.69 to 1.55)	0.001	80.5	0.02
Clinical outcomes nRCT						
Lysholm	13	− 1.53	(− 2.19 to − 0.87)	0.001	NA	NA
ROM	43	− 0.16	(− 0.51 to 0.28)	0.57	NA	NA
HSS	21,23	− 1.72	(− 2.38 to − 1.06)	0.001	80	0.001
KSS	23,31	− 1.52	(− 2.52 to − 0.52)	0.003	85.1	0.01

*AX* Anatomical axia, *PC* posterior cortex, *RCT* randomized controlled trial, *nRCT* non-randomized controlled trial, *NA* not available, *ROM* range of motion, *HSS* Hospital for Special Surgery Knee Score, *KSS* American Knee Society Score, *VAS* Visual Analog Scale Pain Score, *WOMAC* the total of Western Ontario and McMaster University Osteoarthritis Index, *AFTA* anatomical femorotibial angle, *HKA* hip-knee-ankle angle, *MAD* mechanical axis deviation, *MMPTA* mechanical medial proximal tibia angle, *PTSA* posterior tibial slope angle, *CDI* Caton-Deschamps Index, *ISI* Insall-Salvati Index, *BPI* Blackburne-Peel index

For PTS, whether using the posterior tibial cortex or the tibia mechanical axis as a reference line, there were significant differences (*p* < 0.05) between the CWHTO and OWHTO groups at postoperation. Meanwhile, the PTS increased (SMD − 0.71, 95% CI − 1.04 to − 0.37, *p* < 0.05) after OWHTO but decreased significantly after CWHTO (SMD 0.72, 95% CI 0.35 to 1.08, *p* < 0.05) (Figs. 3 and 4).

The patellar height, as evaluated by three indicators (BPI, CDI, and ISI), decreased significantly after OWHTO (*p* < 0.05), except for one subgroup of nRCTs in ISI (Table 3 and Table 4). However, the patellar height after CWHTO demonstrated no significant difference between postoperation and preoperation (*p* > 0.05).

All results of the preoperative comparison and postoperative comparison in the two groups are presented in Table 3. The comparison of preoperative and postoperative outcomes of each group is shown in Table 4. Considering that some included articles (follow-ups: less 1 year) may negatively skew the results, an analysis of subgroups of follow-ups more than 1 year was performed, and the results of subgroups were consistent with the overall results comparing postoperative outcomes of CWHTO and OWHTO (Table 5 and Table 6).

**Table 5:** Radiological results of the preoperative comparison and postoperative comparison between OWHTO and CWHTO after excluding the results of the follow-up within 1 year

Categories and comparison	Studies	Mean difference		*P* value	Heterogeneity	
		Mean	(95% CI)		*I*^2^ (%)	*p* value
CWHTO VS OWHTO in preoperative outcomes						
Radiological outcomes RCT						
HKA	10,24,27,30	0.14	(− 0.11 to 0.39)	0.266	0	0.703
PTSA (AX)	24	− 0.30	(− 0.81 to 0.21)	0.242	NA	NA
ISI	31	0.06	(− 0.49 to 0.61)	0.84	NA	NA
Radiological outcomes nRCT						
PTSA (PC)	12	− 0.18	(− 0.54 to 0.17)	0.314	NA	NA
CDI	42	− 0.25	(− 0.67 to 0.17)	0.24	NA	NA
CWHTO VS OWHTO in postoperative outcomes						
Radiological outcomes RCT						
HKA	10,24, 27,30	0.249	(− 0.01 to 0.503)	0.06	0	0.769
HKA CORRECTION	24, 27	0.15	(− 0.21 to 0.63)	0.40	0	0.35
PTSA (AX)	24	− 1.47	(− 2.04 to − 0.90)	0.001	NA	NA
ISI	31	0.59	(0.02 to 1.15)	0.041	NA	NA
Radiological outcomes nRCT						
PTSA (PC)	12	− 1.72	(− 2.139 to − 1.30)	0.001	NA	NA
CDI	42	1.01	(0.55 to 1.44)	0.001	NA	NA

Postoperative results were compared between the two groups in the lower half of the table

*AX* Anatomical axia, *PC* posterior cortex, *RCT* randomized controlled trial, *nRCT* non-randomized controlled trial, *NA* not available, *HKA* hip-knee-ankle angle, *PTSA* posterior tibial slope angle, *CDI* Caton-Deschamps Index, *ISI* Insall-Salvati Index, *BPI* Blackburne-Peel index

**Table 6:** Preoperative and postoperative imaging outcomes of each group after excluding the results of the follow-up within 1 year

Categories and comparison	Studies	Mean difference		*P* value	Heterogeneity	
		Mean	(95% CI)		*I*^2^ (%)	*P* value
Preoperative VS postoperative results in CWHTO						
Radiological outcomes RCT						
HKA	10,24,27,30	− 3.24	(− 3.64 to − 2.86)	0.001	82.7	0.003
PTSA(AX)	24	0.52	(0.008 to 1.04)	0.046	NA	NA
ISI	31	− 0.06	(− 0.63 to 0.51)	0.84	NA	NA
Radiological outcomes nRCT						
PTSA (PC)	12	0.92	(0.54 to 1.29)	0.003	NA	NA
CDI	42	− 0.21	(− 0.61 to 0.19)	0.31	NA	NA
Preoperative VS postoperative results in OWHTO						
Radiological outcomes RCT						
HKA	10,24,27,30	− 3.02	(− 3.42 to − 2.63)	0.001	92.8	0.001
PTSA (AX)	24	− 0.67	(− 1.18 to − 0.15)	0.012	NA	NA
ISI	31	0.48	(0.06 to 0.64)	0.06	NA	NA
Radiological outcomes nRCT						
PTSA (PC)	12	− 0.58	(− 0.94 to − 0.21)	0.002	NA	NA
CDI	42	0.50	(0.06 to 0.95)	0.026	NA	NA

*AX* Anatomical axia, *PC* posterior cortex, *RCT* randomized controlled trial, *nRCT* non-randomized controlled trial, *NA* not available, *HKA* hip-knee-ankle angle, *PTSA* posterior tibial slope angle, *CDI* Caton-Deschamps Index, *ISI* Insall-Salvati Index, *BPI* Blackburne-Peel index

## Discussion

This meta-analysis of 28 studies indicates that there are no differences (*p* > 0.05) in the function of correction (AFTA and HKA) and main clinical outcomes (HSS, KSS, and VAS) between OWHTO and CWHTO, that PTS increases after OWHTO but decreases after CWHTO (*p* < 0.05), and that patellar height (CDI, BPI, and ISI) decreases (*p* < 0.05) after OWHTO but does not change after CWHTO (*p* > 0.05).

The medial compartment receives a greater load (approximately 60%) in healthy knees than the lateral compartment [43, 44]. If the tibia has some degree of varus deformity, the pressure on the cartilage in the medial compartment will significantly increase beyond the range of tolerance, triggering cartilage wear and inflammation and resulting in medial osteoarthritis [4, 45]. Historically, HTO was first reported by Jackson et al. [46] in the 1960s. The original intention of HTO was to correct tibial varus deformity to properly transfer some stress to the lateral compartment, significantly reducing the pressure in the medial compartment and effectively preventing cartilage wear and relieving pain symptoms. After 50 years of development, HTO has evolved into a much safer, more accurate and more effective surgical procedure for patients with medial compartmental arthritis [37].

Previously, a clinical study including 39 HTO cases [47] presented a reliable result with the survival rate of 82% at 12-year follow-up. If seven patients with bicompartmental osteoarthritis receiving HTO were excluded in this study, it was foreseeable that these results would become better. Therefore, Berman et al. [47] emphasized that considerably strict indications and careful preoperative imaging assessments are critical for HTO. Currently, the best indications for HTO are relatively young, active patients with medial knee osteoarthritis or with a tibial varus greater than 5° [47–49]. Emphatically, most cases included in this meta-analysis are in line with these indications for HTO.

To evaluate the controversial clinical effects between OWHTO and CWHTO, we compared the outcomes according to the included articles [10, 13, 24, 27, 30, 31]. As the results imply, the postoperative results of VAS, HSS, KSS, Lytolom score, and WOMAC were better than the preoperative results in both groups (*p* < 0.05), which indicated that both surgical methods were effective. However, there was no significant difference comparing the postoperative results of CWHTO and OWHTO (*p* > 0.05). Additionally, it should be emphasized that no significant change was found in the range of motion after OWHTO or CWHTO (*p* > 0.05) in this study. Similarly, following up for a minimum of 3 years and 1 year postoperatively, Song et al. [21] and Kim et al. [24] both found that mean maximal flexion was not significantly changed after OWHTO or CWHTO. Furthermore, when evaluating the influence of HTO technique on the performance and results of total knee arthroplasty (TKA) at a mean follow-up of 2 years, Filho et al. [36] indicated that post-TKA range of motion was not different between OWHTO and CWHTO.

Regarding the comparison of radiological results, the correction angle evaluated by H-K-A and AFTA (built between the mechanical axis and anatomical axis of the tibia and femur, respectively), is one of the most important indicators that can influence surgical outcomes [24]. In this study, postoperative H-K-A or AFTA did not show significant differences between OWHTO and CWHTO (*p* > 0.05), while the postoperation H-K-A or AFTA was better (*p* < 0.05) than the preoperation values in both groups, which demonstrated that the correction functions of OWHTO and CWHTO were similar. Three previous studies [13, 41, 44] showed that both CWHTO and OWHTO could provide acceptable correction, which was consistent with our results. Considering the degree of correction, Aglietti et al. [50] indicated that the functional effect of surgery would be maintained at a follow-up of 10 years when keeping the valgus angle (AFTA) at 8 to 15° after HTO. The significant under-correction caused by the incompletely closed gap in CWHTO or the bone deficit in OWHTO could not reduce the medial tibial plateau load, leading to the loss of osteotomy angle and recurrence of varus. Additionally, overcorrection would cause an excessive lateral compartment load, accelerating degeneration of the articular cartilage [51, 52].

The original goal of HTO was to correct deformities of the coronal plane, but unexpected events, the changes in the PTS, occurring in the sagittal position are always inevitable [53, 54]. Our results showed that PTS increases after OWHTO (*p* < 0.05) and decreases (*p* > 0.05) after CWHTO, which is consistent with several comparative studies [16, 18, 23, 24, 26]. Multiple biomechanical and clinical studies attempted to identify the reasons for changes in PTS after HTO [33, 40, 44, 55]. First, surgeons cannot release the posterior soft tissues sufficiently [12], considering to protect muscles and posterior vessels [16, 22]. The other reason is that the triangular shape of the proximal tibia with the apex directed anteriorly makes the center of rotation angulation slightly posterior in the sagittal plane [8, 37, 56]. The demonstrations above cause a less opened posterior gap after OWHTO and a less touched area of the posterior bone after CWHTO.

Flexion and extension activities can be affected by unintended alterations of PTS in the sagittal plane [44, 55]. Second, a linear relationship between tibial slope and tibial translation during unilateral weight-bearing was shown: the greater the angle of slope was, the greater the anterior translation in knees was [12, 55]; therefore, an increased anterior tibial translation after OWHTO can aggravate the load of the anterior cruciate ligament (ACL) [43].

To avoid the undesirable results caused by PTS, many researchers and clinicians have proposed many novel surgical techniques. Nerhus et al. [26] pointed out that PTS after OWHTO increased by a small amount or did not increase at all if the fixation plate was placed close to the posteromedial corner. The osteotomy height and the opening gap at the posteromedial cortex should be higher and broader than those at the tibial tuberosity, which can reduce or avoid changes of PTS pointed out by Giffin et al. [55]. Careful preoperative planning is also quite essential for reducing or preventing changes of PTS in HTOs [26, 38].

Three indicators (BPI, CDI, and ISI) were used to evaluate changes in the patella height. OWHTO reduced the patella height (*p* < 0.05), while statistically significant effects on the patellar height were not found after CWHTO (*p* > 0.05). Using the measurement of CDI, some researchers reported that patellar height decreases by 9 to 16% after OWHTO compared with that before surgery [15, 27, 30, 42], which was similar to our results. However, consistent with our pooled results, Song et al. [21] indicated that patellar height remained unchanged after CWHTO.

The reason for patellar decreasing after OWHTO is that the opening wedge of the medial tibia prolongs the proximal tibia, resulting in a reduction in the height of the patellar tendon attachment site, thus causing a decrease in patellar height. This result occurs because CWHTO circumvents the drawbacks of OWHTO that the patellar height has not changed significantly. Tigani et al. [42] indicated that, according to the measurement of 40 knees after OWHTO, patellar lowering would become significant if the correction of the knee axis exceeded 15°. However, when the tibial tuberosity is left and attached to the proximal tibia [38], the patellar height can maintain unchanged after OWHTO, and the osteotomy site still has excellent blood perfusion due to the cancellous bone surface and is therefore very conducive to bone healing [42].

The main limitations of this systematic review and meta-analysis originate from the data pooled from the included articles. RCTs and nRCTs were both included when comparing OWHTO and CWHTO, especially having a greater caseload in the nRCTs, which may likely lead to bias. Nevertheless, the MINORS scores were acceptable when evaluating the quality of nRCTs, and the results of RCTs and nRCTs were processed respectively in order to reduce heterogeneity. Additionally, the follow-up periods and the internal fixation were diverse, which may affect the final results of the two surgical methods. In some subgroups, we did not find enough data, such as for ISI and BPI.

## Conclusion

This meta-analysis indicates that compared with CWHTO, OWHTO increases the posterior slope, decreases the patellar height, and provides a similar accuracy of correction; however, CWHTO led to a deceased posterior slope and an unchanged patellar height. Therefore, programs should be personalized and customized for the specific situation of each patient.

## Data Availability

Data sharing is not applicable to this article as no datasets were generated or analyzed during the current study.

## Abbreviations

AFTA: Anatomical femorotibial angle
AX: Anatomical axis
BPI: Blackburne-Peel index
CDI: Caton-Deschamps index
HKA: Hip-knee-ankle angle
HSS: Hospital for Special Surgery Knee Score
ISI: Insall-Salvati Index
KSS: American Knee Society Score
MAD: Mechanical axis deviation
MMPTA: Mechanical medial proximal tibia angle;
NA: Not available
nRCT: Non-randomized controlled trial
PC: Posterior cortex
PRISMA: Systematic Reviews and Meta-Analyses
PTSA: Posterior tibial slope angle
RCT: Randomized controlled trial
ROM: Range of motion
VAS: Visual Analog Scale Pain Score
WOMAC: The total of Western Ontario and McMaster University Osteoarthritis Index
